# Pediatric mixed headache -The relationship between migraine, tension-type headache and learning disabilities - in a clinic-based sample

**DOI:** 10.1186/s10194-016-0625-x

**Published:** 2016-04-22

**Authors:** Jacob Genizi, Amal Khourieh Matar, Mitchell Schertz, Nathanel Zelnik, Isaac Srugo

**Affiliations:** Department of Pediatrics, Bnai Zion Medical Center, Haifa, Israel; Pediatric Neurology Unit, Bnai Zion Medical Center, Haifa, Israel; Child Development & Pediatric Neurology Service, Meuhedet – Northern Region, Haifa, Israel; Pediatric Neurology Unit, Carmel Medical Center, Haifa, Israel; Bruce and Ruth Rappaport Faculty of Medicine, Technion, Haifa, Israel

**Keywords:** Migraine, TTH, Children, Epidemiology

## Abstract

**Background:**

Headache is a common complaint among children. The most common primary headache syndromes in childhood are migraine and TTH. However many times they seem to overlap. The purpose of our study was to assess the relationship between pediatric migraine, tension-type headache (TTH) and learning disabilities.

**Methods:**

Children presenting with headache to three pediatric neurology clinics in the last 5 years were assessed. Two hundred sixty-two children, 5–18 years of age, who met the criteria for migraine were included.

**Results:**

Of 262 children (54 % female) who had migraine, 26.2 % had migraine with aura. 59 children (22.5 % of the full sample) reported also having headaches that met the criteria for episodic TTH/mixed headaches. Females were more than 2.8 times more likely to experience mixed headaches than males (OR: 2.81, 95 % CI: 1.43–5.54; *p* <.003). Multiple logistic regression analysis revealed that older age (*p* <0.02), family history of aura (*p* <.02), and (lack of) TTH (*p* <.003) were significant predictors of aura, whereas gender was not significant (*p* >0.20). Children who had migraine with aura were less likely to have mixed headaches than children who did not have aura (OR: 0.26, 95 % CI: 0.11–0.63; *p* <.003). Children with mixed headaches were 2.7 times more likely to have a learning disability than children with migraine alone.

**Conclusions:**

Episodic TTH and migraine without aura (mixed headaches) in children might be part of a continuum, which can explain the high incidence of their co-occurrence as opposed to migraine with aura. Children with mixed headaches have a higher incidence of learning disability compare to those with migraine alone.

## Background

Migraine and tension-type headache (TTH) are by far the most common types of headache in children [1]. The prevalence of migraine increases from 3 % in the preschool years to 4–11 % by the elementary school years, and up to 8–23 % during the high-school years [2]. The prevalence of TTH is 9.8–18 % [3–5]. The relationship between migraine and TTH is debated. The ICHD-II and III beta criteria [6, 7] differentiate between the two, viewing them as wholly different syndromes. However, changes in the symptoms reported as children age is a complicating issue for differential diagnosis which supports the continuum model [8–10]. Battistella [4] reported that in 14 % of young children diagnosed as having migraine without aura, symptoms evolved in adolescence to those of episodic tension-type headache. Nachit-Ouinekh [11] also reported instability in the formal assignment of a migraine headache and TTH diagnosis and a drift between headache types. In daily practice, migraine and TTH share common epidemiological and clinical features which make it difficult to differentiate between them. Indeed some practitioners claim that the majority of migraineurs also have TTH [4].

The aim of the present study was to assess the prevalence and features of children presenting with simultaneous migraine and TTH and the association of learning disabilities as a trigger.

## Method

### Study population and design

We retrospectively reviewed medical records of children and adolescents who were referred by their primary physician for neurological assessment due to headaches to the outpatient pediatric neurology clinics at the Bnai Zion Medical Center, Carmel Medical Center and Meuhedet Medical Services, all in the city of Haifa, during the years 2009–2014. Children and adolescents aged 5–18 years meeting diagnostic criteria for migraine according to the 2004 ICHD-II (IHS-2) were included [6]. Children with chronic headache were excluded. During their initial and follow-up visits to the pediatric neurology clinic, all patients and parents were interviewed using a semi-structured format according to the HIS criteria and were asked to complete a written questionnaire and to write a headache diary. Both the interview the questionnaire and the headache diary included questions regarding demographics, the patient’s and family’s medical history, and headache history (age at onset, location, quality, frequency, duration of episodes, aura, and associated symptoms). Children who complained of learning difficulties were given a formal psycho-educational assessment. The study was approved by the Bnai Zion IRB # 89/14.

### Statistical evaluation

Comparisons were performed via chi-square tests or Fisher’s exact test where appropriate for the categorical data and via independent t-tests for the continuous variables. Odds ratios and their 95 % confidence intervals were computed. Significance was considered to be *p* <0.05. Multiple logistic regression analysis was performed using gender, age and family history of migraine as main effects. Following this, stepwise logistic regression was used to evaluate two-way interactions as potential predictors. Statistical analysis was performed using SPSS software version 21 (SPSS, Chicago, IL). The study was approved by the local Helsinki committee.

## Results

During the study period, 262 children (mean age 12 years, SD = 2.9) were diagnosed with migraine headache. Of the total, 138 were female (54 %) and 124 were male (46 %). Sixty-eight children (26.2 %) experienced aura (Table 1). Careful history revealed that 59 children (22.5 %) met the criteria for episodic TTH/mixed headaches in addition to their migraine headaches; of these, 43 (73 %) were female and 16 (27 %) were male. Females were more than 2.8 times as likely to experience mixed headaches compared to males (OR: 2.81, 95 % CI: 1.43–5.54; *p* <.003).

**Table 1:** Demographic and clinical data (number and % or mean ± SD) of children with Migraine

	Aura (*N* = 68)	No Aura (*N* = 192)	*p*
Gender			.01
Male	23 (18.9 %)	99 (81.1 %)	
Female	45 (32.6 %)	93 (67.4 %)	
Age (range)	13.1 ± 2.9 (5–18)	11.5 ± 3.2 (3–21)	<.001

### Mixed headaches and migraine with aura

Multiple logistic regression analysis revealed that older age (*p* <0.02), family history of aura (*p* <.02) and (lack of) TTH (*p* <.003) were significant predictors of aura. Gender was not significant (*p* >0.20). Children who had mixed headaches were 83 % less likely to experience migraine with aura than children without mixed headaches (OR: 0.17, 95 % CI: 0.05–0.54; *p* <.003). Conversely, children who had migraine with aura were 74 % less likely to have mixed headaches than children who did not have aura (OR: 0.26, 95 % CI: 0.11–0.63; *p* <.003) (Table 2). There was no association between family history of migraine or age and mixed headaches.

**Table 2:** The relationship between TTH headaches and Migraine with Aura

	TTH (*N* = 59)	No TTH (*N* = 182)	*p*
Migraine with Aura			.03
Yes	10 (14.9 %)	57 (28.2 %)	
No	49 (85.1 %)	125 (71.8 %)	

### Mixed headaches and learning disabilities

We found a significant association between learning disabilities, diagnosed using a formal psycho-educational assessment, and mixed headaches (*p* <.003) (Table 3). Children with mixed headaches were nearly 2.7 times more likely to have a learning disability than children with migraine alone (OR: 2.69, 95 % CI: 1.37–5.28, *p* <0.003).

**Table 3:** The relationship between mixed headaches and learning disabilities

	Migraine		
	TTH (*N* = 59)	No TTH (*N* = 182)	*p*
Learning disabilities			.003
Yes	26 (52.0 %)	37 (28.7 %)	
No	24 (48.0 %)	92 (71.3 %)	
Unknown	9 (15.2 %)	53 (29.1 %)	

## Discussion

We report on the characteristics of a large cohort of children and adolescents with migraine. Our cohort included more females (54 %), and a sizable percentage of children (26 %) experienced aura. These findings are in line with previous reports [12, 13]. The children were asked if they experienced additional headaches besides those compatible with migraine, and 24 % reported having headaches that fulfil the criteria for episodic TTH. Among the group reporting mixed headaches, the percentage of females was even higher (73 %).

The 22 % overlap between TTH and migraine observed in our study should be considered against the backdrop of previous studies. Both Silberstein [14] among adults and Viswanathan [8] in children considered migraine and TTH to be closely related entities which diverge only in severity. In support of this argument, a number of studies among adults (the Spectrum study [15]) and children [16, 17] found an evolution to migraine headache among patients with TTH. Turkdogan found that 58 % of children with migraine reported TTH features and 68 % of children with episodic TTH had migraine-type features; however, he considered this an overlap between features of migraine and TTH and vice versa, rather than a syndrome combining migraine and TTH [10]. The 24 % overlap of TTH and migraine as was demonstrated in our study can be explained in few different ways. If we are not willing to agree with Silberstein and Viswanathan that migraine and TTH are a continuum, it might be due to wrong diagnosis. Battistella reported that 14 % of young children diagnosed as migraine without aura had evolved in adolescents into episodic tension-type headache. But he reported that none of children with TTH evolved into migraine even without aura?! Another explanation might be the common triggers. Pediatric migraine and TTH are both triggered mainly by stress [5, 18] which might explain the stimulus presentation.

In this regard, the current findings on gender are also worth noting. The increased incidence of all migraine among females in comparison to males is well known [3, 18]. Laurell, in her population-based survey, reported a higher percentage of females in TTH as well as in migraine [3]. Lyngberg, in a large epidemiologic study, reported a male:female ratio of 1:6 in migraine, but only 1:3 in episodic tension-type headache [19]. In our study, the proportion of females was substantially higher among the mixed-headache group (migraine + TTH) than among those with only migraine. These findings again raise the question, are mixed headaches really a different syndrome? Of course, it is also possible that the answer lies in the common trigger for migraine and TTH, namely emotional stress. More specifically, does stress trigger these conditions, and in particular migraine without aura, equally in males and females? If stress is the main trigger of migraine in females but not in males, we can expect females to have greater comorbidity of migraine and TTH, which is also triggered by stress. In males, only a small number should have mixed headaches, as most migraines in this group will be caused by something other than stress.

In our study we found that children with mixed headaches were less likely to have migraine with aura. Given that it is easier to differentiate between migraine with aura and TTH, this finding may simply reflect misdiagnosis of TTH as migraine without aura. Alternatively, it may be that TTH is related to migraine without aura and both are part of a continuum, while migraine with aura is a different disease. This theory is bolstered by our findings that the predictors of migraine with aura include family history of aura but not of migraine alone, and an absence of TTH.

Finally, the connection between mixed headaches and learning disabilities is worthy of mention. In our previous study [13] we found that learning disabilities were more common among children with migraine compared to children with TTH alone. D’Andrea [20] and Waldie [21] reported impairment in memory in children with migraine, with normal performance in reading, motor and spatial tasks. Parisi [22] found significant differences between the headache (Migraine and TTH) and control groups in the mean total intelligence quotient and verbal intelligence quotient scores, and a negative correlation between the total intelligence quotient score and the age at headache onset. Haverkamp [23] found no significant difference in sequential and simultaneous information processing when comparing the cognitive performance of children with migraine to their healthy siblings. Previous studies have found that adolescents with headaches tend to be highly motivated over-achievers [24].

Parisi [22] findings that headache starting at early age causes more intelligence deficit do not confirm that migraine headaches and cognitive impairment both arise in the early childhood. It may suggest, however, that early age at onset and a high frequency of headache attacks are associated with cognitive impairment probably owing to the immaturity of the central nervous system at young age. Among migraine patients, the involvement of cognitive function might be also related to cortical areas, such as the frontal and prefrontal areas, as a result of poor sleep [25], and sub-cortical areas, as a result of iron accumulation in deep brain nuclei [26]. In children with TTH, Parisi [22] found that the verbal comprehension subtest score alone was significantly lower than in the control group.

However, such studies did not distinguish between migraine with and without aura. In the present study we found a significant association between learning disabilities and mixed headaches, but not migraine with aura. These findings cannot be explained on the grounds that stress at school is a trigger for headaches, because this is true for both migraine with and without aura. We regard our findings, including the high percentage of mixed headaches in our study, as further evidence that migraine without aura and TTH exist along a continuum and together create a different syndrome, while migraine with aura a has a unique heritage and characteristics.

### Limitations

Our study is a retrospective, clinic-based study that might not represent the general pediatric population. In addition, subjects were drawn from three different neurology clinics which might have differed in their clinical approach, although using the same IHS criteria.

## Conclusion

We report that children with migraine without aura frequently have concomitant TTH in what is termed mixed headaches. Furthermore, this group has a significant association with learning disabilities. These findings support the theory that migraine without aura and TTH are part of a headache continuum.
